# Ciliary phenotyping in renal epithelial cells in a cranioectodermal dysplasia patient with *WDR35* variants

**DOI:** 10.3389/fmolb.2023.1285790

**Published:** 2023-12-12

**Authors:** Joanna Walczak-Sztulpa, Anna Wawrocka, Łukasz Kuszel, Paulina Pietras, Marta Leśniczak-Staszak, Mirosław Andrusiewicz, Maciej R. Krawczyński, Anna Latos-Bieleńska, Marta Pawlak, Ryszard Grenda, Anna Materna-Kiryluk, Machteld M. Oud, Witold Szaflarski

**Affiliations:** ^1^ Department of Medical Genetics, Poznan University of Medical Sciences, Poznan, Poland; ^2^ Department of Histology and Embryology, Poznan University of Medical Sciences, Poznan, Poland; ^3^ Department of Cell Biology, Poznan University of Medical Sciences, Poznan, Poland; ^4^ Department of Ophthalmology, Poznan University of Medical Sciences, Poznan, Poland; ^5^ Department of Nephrology, Kidney Transplantation, and Hypertension, The Children’s Memorial Health Institute, Warsaw, Poland; ^6^ Department of Human Genetics, Radboud University Medical Center, Nijmegen, Netherlands; ^7^ Department of Human Genetics, Donders Institute for Brain, Cognition, and Behaviour, Radboud University Medical Center, Nijmegen, Netherlands

**Keywords:** cranioectodermal dysplasia, CED, ciliopathy, second-stage renal disease, hURECs, *WDR35*

## Abstract

**Background:** Cranioectodermal dysplasia (CED) is a skeletal autosomal recessive ciliopathy. The characteristic clinical features of CED are facial dysmorphisms, short limbs, narrow thorax, brachydactyly, ectodermal abnormalities, and renal insufficiency. Thus far, variants in six genes are known to be associated with this disorder: *WDR35*, *IFT122*, *IFT140*, *IFT144*, *IFT52*, and *IFT43*.

**Objective:** The goal of this study was to perform cilium phenotyping in human urine-derived renal epithelial cells (hURECs) from a CED patient diagnosed with second-stage chronic kidney disease (CKD) and three unrelated and unaffected pediatric controls.

**Methods:** Genetic analysis by *WDR35* screening was performed in the affected individual. Cilium frequency and morphology, including cilium length, height, and width, were evaluated by immunofluorescence (IF) experiments in hURECs using two markers visualizing the ciliary axoneme (Acet-Tub and ARL13B) and the base of the cilium (PCNT). The IF results were analyzed using a confocal microscope and IMARIS software.

**Results:** *WDR35* analysis revealed the presence of a known nonsense p. (Leu641*) variant and a novel missense variant p. (Ala1027Thr). Moreover, comparative genomic hybridization analysis showed that the patient carries a microdeletion on chromosome 7q31.1. Ciliary phenotyping performed on hURECs showed morphological differences in the patient’s cilia as compared to the three controls. The cilia of the CED patient were significantly wider and longer.

**Conclusion:** The obtained results suggest that CED-related second-stage CKD might be associated with cilia abnormalities, as identified in renal epithelial cells from a CED patient harboring variants in *WDR35*. This study points out the added value of hURECs in functional testing for ciliopathies.

## 1 Introduction

Cranioectodermal dysplasia (CED, Sensenbrenner syndrome) is a rare autosomal recessive heterogeneous condition. It belongs to a group of disorders known as ciliopathies and is associated with defective cilia structure and function. To date, more than 30 ciliopathies and at least 247 associated disease genes have been described in the literature. It is known that various factors, including genetic background, protein function, cell type, and modifier genes, account for clinical heterogeneity in patients with ciliopathies (Mill et al., 2023). Cranioectodermal dysplasia is one of the ciliary chondrodysplasia. To date, six intraflagellar transport (IFT) genes (*IFT122*, *WDR35*, *IFT140*, *IFT144*, *IFT43*, and *IFT52*) have been associated with this disorder. CED is most frequently (approximately 50% of the described patients) caused by pathogenic variants in *WDR35* (#OMIM 613610, CED2). Variants in this gene have also been identified in individuals with short-rib polydactyly syndrome (SRPS), Ellis–van Creveld syndrome (EvC), and Jeune asphyxiating thoracic dystrophy (JATD) (Mill et al., 2011; Caparros-Martin et al., 2015; Zhang et al., 2018a). WDR35 protein, together with IFT144, IFT140, IFT139, IFT122, and IFT43, belongs to complex A of the retrograde intraflagellar transport (IFT) machinery. The main role of IFT is participation in cilia assembly, disassembly, and maintenance. The IFT-A complex is driven by dynein and carries particles along the axonemal microtubules from the tip of the cilium back to the cell body, while the IFT-B complex is driven by kinesin in the opposite direction from the cell body to the tip of the cilium (anterograde IFT) (Pazour et al., 2020; McConnachie et al., 2021). The primary cilia are present on the surface of most vertebrate cells, such as the embryonic node, kidneys, liver, heart, and retina. Moreover, the cilia are structures that play a crucial role in signaling pathways, including Hedgehog, Wnt, GPCR, and BMP, which are important for proper organ development and homeostasis. The primary cilia can transduce extracellular signals to regulate the process of migration, differentiation, and proliferation in a cell type-specific manner. Defects in cilia structure and function influence a variety of signaling pathways in a wide range of ciliated organs. The importance of the cilia in health and disease is evident from the broad phenotypic spectrum seen in ciliopathies. One of the organs in which the cilia play a crucial role in the organogenesis and maintenance of epithelial cell differentiation and proliferation is the kidney. In the kidney, the cilia are mainly present in the proximal tubules, distal tubules, and collecting ducts, and cilium dysfunction is related to the early stages and progression of renal diseases (Bai et al., 2022). Renal insufficiency is a major cause of morbidity and mortality in CED patients (Tan et al., 2021). The occurrence of renal insufficiency in CED-affected individuals with *WDR35* variants is very high and accounts for approximately 93% of reported patients (25 out of 27 CED patients). Kidney dysfunction often leads to end-stage renal disease (ESRD), and 10 out of 25 patients with CED suffering from kidney insufficiency received a kidney transplant. To learn more about cilium function and its role in kidney dysfunction, methods have been developed to obtain hURECs from patients and controls in a non-invasive way. This allows researchers to perform functional experiments in patient-derived cells that originate from the affected organ of interest. Furthermore, hURECs can be used to study the cilium phenotype in patients diagnosed with a ciliopathy, such as Mainzer–Saldino syndrome and Joubert syndrome, as previously described in the literature (Srivastava et al., 2017; Oud et al., 2018). Here, we report on a 5-year-old female patient diagnosed with CED and second-stage CKD. Upon detection of two likely causative variants in the *WDR35* gene, ciliary phenotyping was performed in patient-derived URECs. These experiments revealed morphological differences in the cilia present in the CED patient compared to the three controls.

## 2 Materials and methods

### 2.1 Clinical examination

Clinical examinations were performed by clinical geneticists, ophthalmologist and nephrologists at the Department of Medical Genetics, at the Department of Ophthalmology, Poznan University of Medical Sciences, and the Department of Nephrology, Kidney Transplantation, and Hypertension, The Children’s Memorial Health Institute in Warsaw, respectively.

### 2.2 Collection of samples

Genomic DNA of the patient and parents was extracted from whole blood and used for *WDR35* screening, array comparative genomic hybridization (aCGH) analysis, and quantitative real-time polymerase chain reaction (qPCR) analysis. Urine samples were collected from the patient and three healthy controls and used to analyze the ciliary phenotype. This study was approved by the Bioethics Committee at Poznan University of Medicine Sciences in compliance with the Good Clinical Practice and Polish law. Written informed consent was obtained from the parents. The research was covered by appropriate insurance for medical experiments.

### 2.3 Genetics analysis

#### 2.3.1 *WDR35* screening

All coding and flanking intronic regions, including 20 bp upstream and downstream of exons of *WDR35*, were amplified by PCR reaction and sequenced by the Sanger method. The sequencing results were aligned with the reference sequence of *WDR35* (NM_001006657.1).

#### 2.3.2 Array comparative genomic hybridization

aCGH analysis was performed on 8 × 60 k SurePrint G3 CGH ISCA v2 from Agilent according to the provided protocol.

#### 2.3.3 qPCR

In order to confirm the presence of 7q31 deletion detected by aCGH, a qPCR was performed using a ViiA™ 7 real-time thermal cycler (Applied Biosystems). Family members were analyzed to determine the segregation of the variant within the family. For this purpose, seven pairs of primers were designed, four of which spanned the deletion region, and three more were complementary to the regions adjacent to the deletion, both from the 5′ and 3′ ends. All reactions were run in triplicate. Target sequences were normalized to albumin. The comparative ΔΔCt method was used to determine the gene copy number and calibrated using DNA from a healthy control.

### 2.4 Urine-derived renal epithelial cell isolation and culture

Midstream urine samples (50–100 mL) were collected and kept on ice for up to 4 h. Cells were retrieved from the urine by centrifugation, washed once with PBS containing 0.1 mg/mL Primocin (InvivoGen), and cultured in 2 mL of primary medium consisting of a 1:1 ratio of Dulbecco’s Modified Eagle medium (DMEM, Cytiva):Ham’s F-12 Nutrient Mix (Gibco) containing 10% FBS (Invitrogen), 0.1 mg/mL Primocin (InvivoGen), and 1 x REGM SingleQuots (Lonza) in a 12-well plate. Cells were incubated at 37°C with 5% CO_2_. 1 mL of primary medium was added 24, 48, and 72 h after cell isolation. At 96 h after cell isolation, 2 mL of the culture medium was replaced with 2 mL of urine-derived renal epithelial cell (UREC) proliferation medium containing REBM basal medium (Lonza), 1x REGM SingleQuots, and 0.1 mg/mL Primocin (InvivoGen). Every day, 2 mL of the culture medium was replaced with fresh UREC proliferation medium until the URECs were visible and reached approximately 80% confluence.

### 2.5 Ciliary phenotyping in URECs

To determine the ciliary phenotype, we studied hURECs obtained from the CED patient (P1: 4.5-year-old female) and three unrelated and unaffected controls (C1: 4.5-year-old female; C2: 9.5-year-old male; and C3: 9.5-year-old female). hURECs were analyzed for cilium frequency and morphology by immunofluorescence experiments. hURECs were cultured (P1 at passage no. 3; C1–C3 at passage no. 2) on a ibidi 8-well chamber (ibidi GmbH) until they reached ∼80% confluence. Subsequently, the cells were serum starved for 48 h using the UREC proliferation medium without FBS. After fixation with 2% PFA (Sigma), permeabilization with 1% Triton, and blocking with 2% BSA (Sigma), the cells were incubated with primary antibodies targeting acetylated-alpha-tubulin (Acet-Tub) at 1:1000 (mouse monoclonal T6793, Sigma-Aldrich), ADP-ribosylation factor-like protein 13B (ARL13B) at 1:500 (rabbit polyclonal, Proteintech Group), and pericentrin (PCNT) at 1:1000 (mouse monoclonal, Abcam Cambridge), followed by incubation with fluor-labeled secondary antibodies: anti-mouse Alexa Fluor 568, anti-rabbit Alexa Fluor 488, and anti-mouse Alexa Fluor 647 from Thermo Fisher Scientific. The coverslips were embedded in VECTASHIELD PLUS Antifade Mounting Medium with DAPI (Vector Laboratories).

### 2.6 Microscopic analysis

#### 2.6.1 Imaris 3D reconstruction and parametric analysis

The measurements were carried out on URECs at passages 2–3. Z-stack images were acquired using an Olympus FV10i confocal laser scanning microscope and processed with Imaris 7.4.2 (Bitplane, UK) for 3D reconstruction. The volume and sphericity of the nucleus (DAPI), cilia (Acet-Tub and ARL13B), and pericentrin (PCNT) were automatically calculated based on Z-stacks composed of individual images. The cilia axis length was then calculated using three parameters (A, B, and C) multiplied by 2, following the manufacturer’s instructions (http://www.bitplane.com/download/manuals/ReferenceManual9_2_0.pdf). Axis A was the length of the shortest principal axis, axis B was the length of the second longest principal axis, and axis C was the length of the longest principal axis (Supplementary Figure S1). The analysis included a minimum of 100 cilia per individual, and the cilium frequency analysis included 100 cells per individual.

### 2.7 Statistical analysis

For parametric analysis of acetylated-alpha-tubulin (Acet-Tub), ADP-ribosylation factor-like protein 13B (ARL13B), pericentrin (PCNT) volume, and cilia axes A, B, and C length, a two-tailed *t*-test was used to assess statistical significance. For each tested sample, the percentage of the nuclei with cilia was analyzed using one-way ANOVA with Tukey’s multiple comparison tests, employing a single pooled variance. For group analysis, the percentage of nuclei with cilia was compared using a two-tailed *t*-test. Statistical analysis was performed using GraphPad Prism 10 for Windows (ns: not significant, *p < 0.05, **p < 0.01, ***p < 0.001, and ****p < 0.0001).

## 3 Results

### 3.1 Clinical description

**FIGURE 1:**
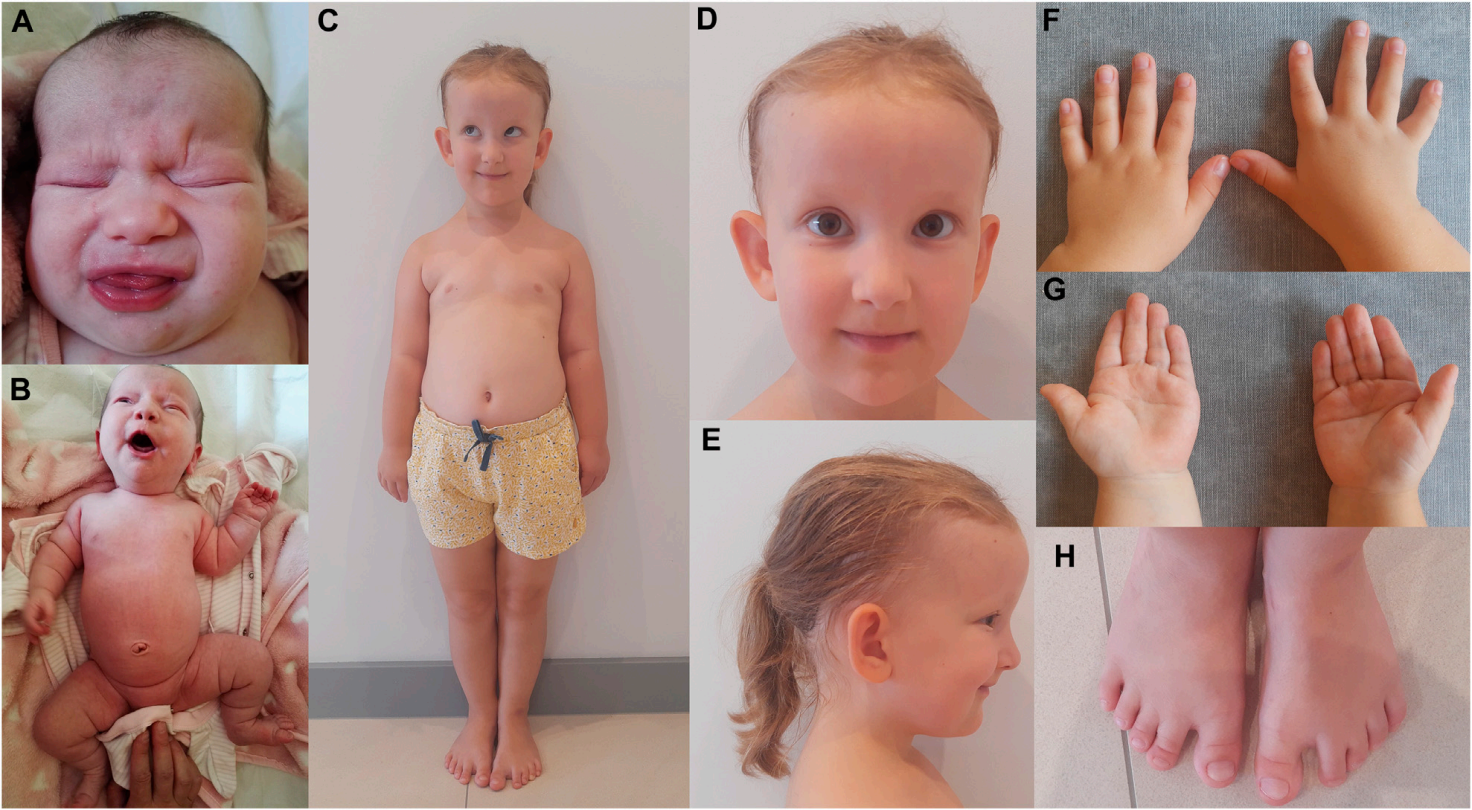
CED patient after birth **(A, B)** and at the age of 5 years **(C–H)**. Disproportionately high forehead **(A, C–E)**, frontal bossing **(C–E)**, dolichocephaly **(E)**, upslanting palpebral fissures **(C, D)**, hypertelorism and broad nasal bridge **(A, D)**, sparse and fine scalp hair and large and protruding ears **(C–E)**, shortening of limbs with rhizomelia and a short and narrow thorax **(B, C)**, broad hands and brachydactyly of the hands **(F, G)** and feet **(H)**, single palmar crease **(G)**, and short and broad toes, short nails, soft-tissue 2**–**3 syndactyly, and a gap between the hallux and second toe **(H)**.

Here, we describe a 5-year-old female CED patient. She was the first child of unrelated and apparently healthy parents. The delivery was by caesarian section in the 41st week of gestation. The birth weight was 4,400 g (>97th percentile), birth length was 53 cm (97th percentile), and Apgar score was 10. Prenatal ultrasound examination revealed the presence of abnormalities, including an increased nuchal translucency (NT = 2.9 mm at 12 weeks and 1 day of gestation and NT = 4.6 mm at 14 weeks and 5 days of gestation), which led to invasive prenatal testing (amniocentesis) at 15 weeks and 6 days of gestation. The adjusted risk for trisomy 21 based on maternal age (35 years) and USG examination (increased NT) was estimated to be 1:76. Aneuploidy of chromosomes 13, 18, 21, X, and Y was excluded by the QF-PCR method. At the age of 2 months, USG analysis showed bilateral kidneys with increased parenchyma echogenicity and lack of corticomedullary differentiation. At the age of 4 months, the head circumference (OFC, occipital frontal circumference) was 40 cm (25–50 percentile), height was 63 cm (50–85 percentile), and weight was 6 kg (50–75 percentile). Clinical examination by a clinical geneticist revealed a high forehead, frontal bossing, dolichocephalic head shape, upslanting palpebral fissures, hypertelorism and a broad nasal bridge, sparse and fine scalp hair, large and protruding ears, shortening of limbs with rhizomelia, short and narrow thorax, broad hands, brachydactyly of the hands and feet, single palmar crease, short and broad toes, short nails, soft-tissue 2–3 syndactyly, and a gap between the hallux and second toe (Figures 1A–H). Based on these clinical features, the patient was diagnosed with cranioectodermal dysplasia (OMIM 613610). The patient was able to sit independently at the age of 8 months and walked with no assistance at 13 months of age. She started speaking at the age of 18 months, and her happy friendly behavior was noted. Nephrological examination at the age of 4.5 years revealed that the patient presented with stage-II CKD with a current estimated glomerular filtration rate (eGFR) of 78 mL/min/1.73m^2^ (criterion for CKD stage-II CKD: eGFR 89–60 mL/min/1.73m^2^). The serum creatinine concentration increased within the 4 months from the previous examination 0.39 mg/dL to 0.64 mg/dL (reference ranges: 44–0.64 mg/dL). Renal ultrasound examination showed normal-sized kidneys, with increased echogenicity of cortical parenchyma, without the presence of cysts in the kidney cortex or in the medulla. There was a discrete dilatation of the right kidney’s pelvis (4 mm diameter). Renal blood flow was normal in Doppler USG. Ultrasound scan of the liver, spleen, and pancreas revealed no abnormalities. An ophthalmological examination performed at the age of 5 years showed full visual acuity in both eyes. The anterior structures of the eye and eye fundus were normal. Moreover, inner and outer canthal distances were within normal limits. Measurement of cycloplegic refractive error revealed slight hypermetropia in both eyes (RE/LE +1.5 Dsph). Electroretinography (standard International Society for Clinical Electrophysiology—ISCEV protocol, skin electrodes) revealed normal retinal function.

### 3.2 Genetic analysis

Molecular genetic analysis of *WDR35* (NM_001006657.1) by sequencing revealed the presence of compound heterozygous variants. The two variants included a previously described nonsense variant in exon 18 (c.1922T>G; p. (Leu641*); rs199952377) and a novel missense variant in exon 26 [c.3079G>A; p. (Ala1027Thr)]. Segregation analysis revealed that the mother and father are each carriers of p. (Ala1027Thr) or p. (Leu641*), respectively. The p. (Leu641*) variant has previously been reported in eight families diagnosed with CED and two families with JATD (Hoffer et al., 2013; Li et al., 2015; Zhang et al., 2018a; Brndiarova et al., 2021; Walczak-Sztulpa et al., 2021; Walczak-Sztulpa et al., 2022). The frequency of this variant in the Genome Aggregation Database (GnomAD, 22.06.2023) is 0.000204 (51/250358 alleles), which suggests that this variant is rare in the general population. This variant was classified as pathogenic based on the ACMG classification system. Variant p. (Ala1027Thr) was classified as a variant of uncertain significance (VUS) according to the ACMG guidelines. This variant in exon 26 is a novel missense change absent from GnomAD, suggesting that it is uncommon in the general population. Although the variant does not reach the official ACMG criteria to be likely pathogenic, we consider it highly suspicious and probably casual for the phenotype of this CED patient.

aCGH analysis on DNA from the CED patient revealed the presence of an approximately 250 kb deletion on the long arm of chromosome 7q31.1. The microdeletion was confirmed by qPCR and was also present in the DNA of the apparently healthy mother.

### 3.3 hUREC culture

**FIGURE 2:**
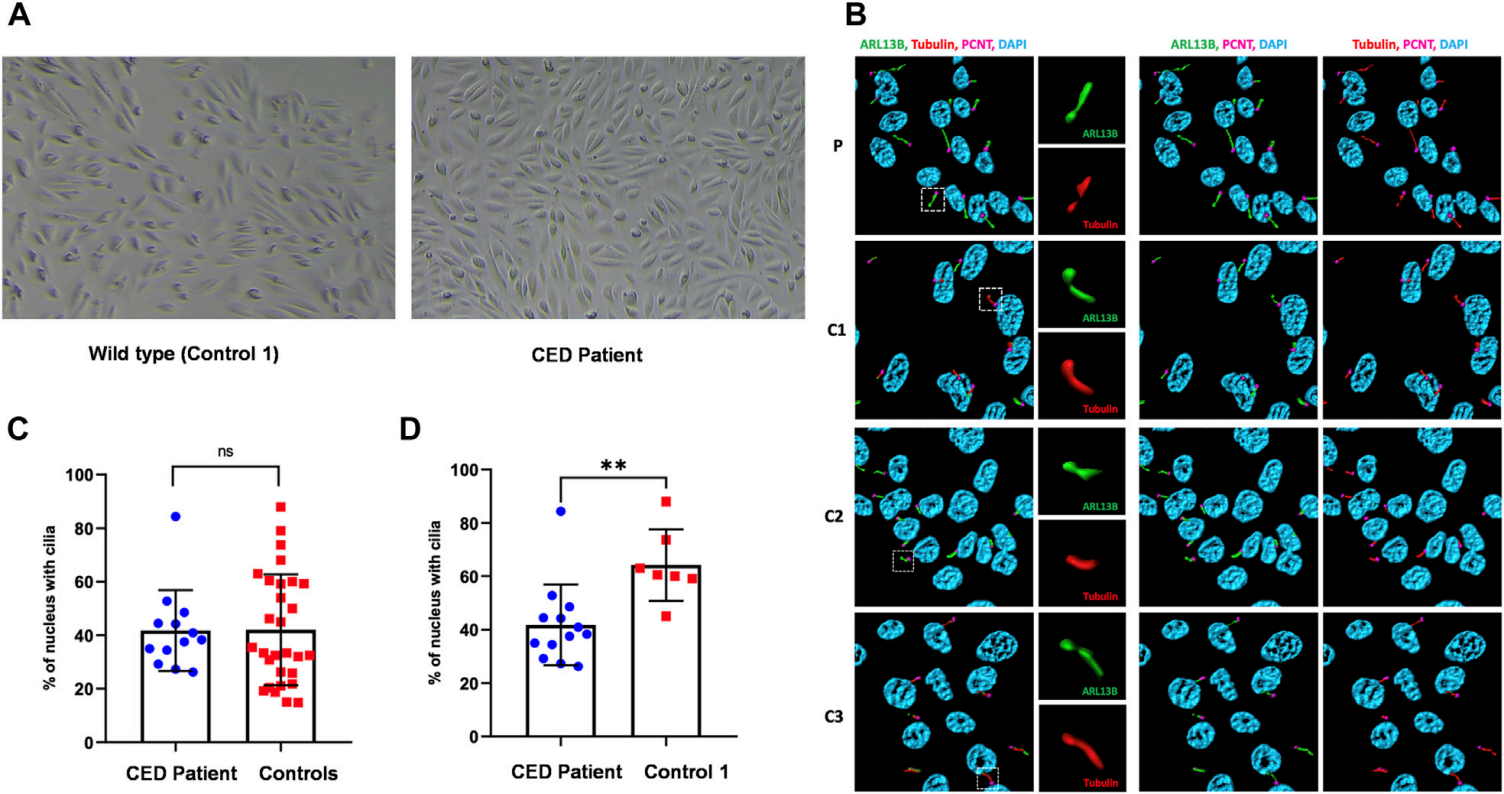
Renal epithelial cells (URECs) derived from control 1 and CED patient with *WDR35* variants presenting a typical cobblestone epithelial cell layer when grown in 2D culture **(A)**. Human URECs following IF imaging using anti-ARL13B (green) for the ciliary membrane, anti-acetylated-tubulin (red) for the ciliary axoneme, PCNT (pink) to mark the base of the cilium, and DAPI (blue) for the nuclei are shown. P, CED patient; C1, control 1; C2, control 2; and C3, control 3 **(B)**. The IF experiments revealed no significant differences in ciliogenesis between the CED patient and the combined controls **(C)**. There was, however, a statistical difference (*p* < 0.01) between the CED patient and control 1, a sex- and age-matched control **(D)**.

First colonies of hURECs were visible approximately 6 days after sample/urine collection in all four samples (CED patient and three controls). The cells from the CED patient appeared to have a higher proliferation rate and were growing faster compared to those of the controls (Figure 2A).

### 3.4 Ciliary phenotyping

**FIGURE 3:**
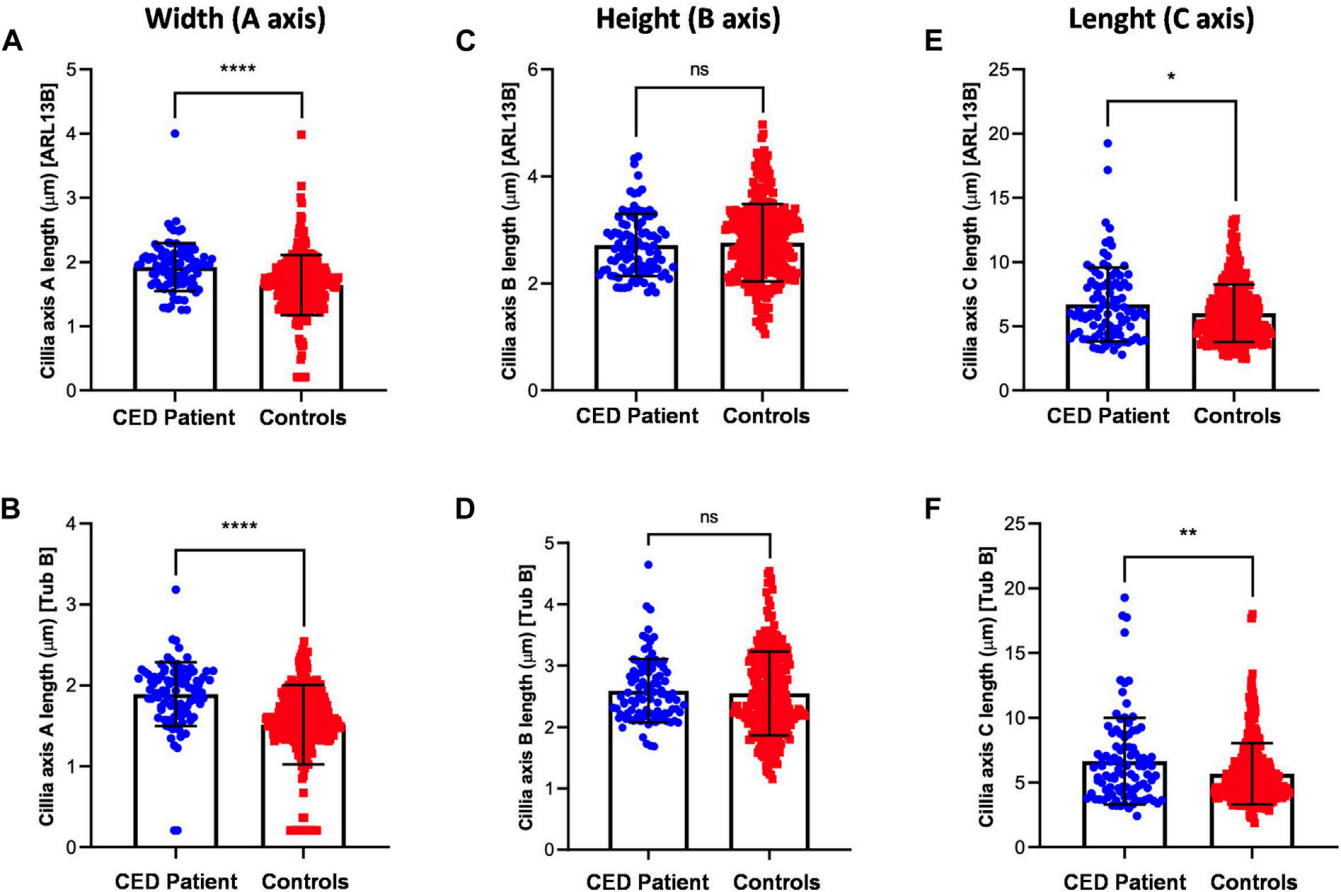
Cilium length, height, and width were analyzed using two antibodies specific for Acet-Tub and ARL13B. Examination of the cilia width (A axis) revealed highly significant differences between the CED patient and controls for both markers (Acet-Tub and ARL13B: *p* < 0.0001) **(A, B)**. Evaluation of the ciliary height showed no differences between the CED patient and controls **(C, D)**. Analysis of the ciliary length revealed longer cilia in the CED patient as compared to controls for both proteins (Acet-Tub: *p* < 0.01 and ARL13B: *p* < 0.05) **(E, F)**.

**FIGURE 4:**
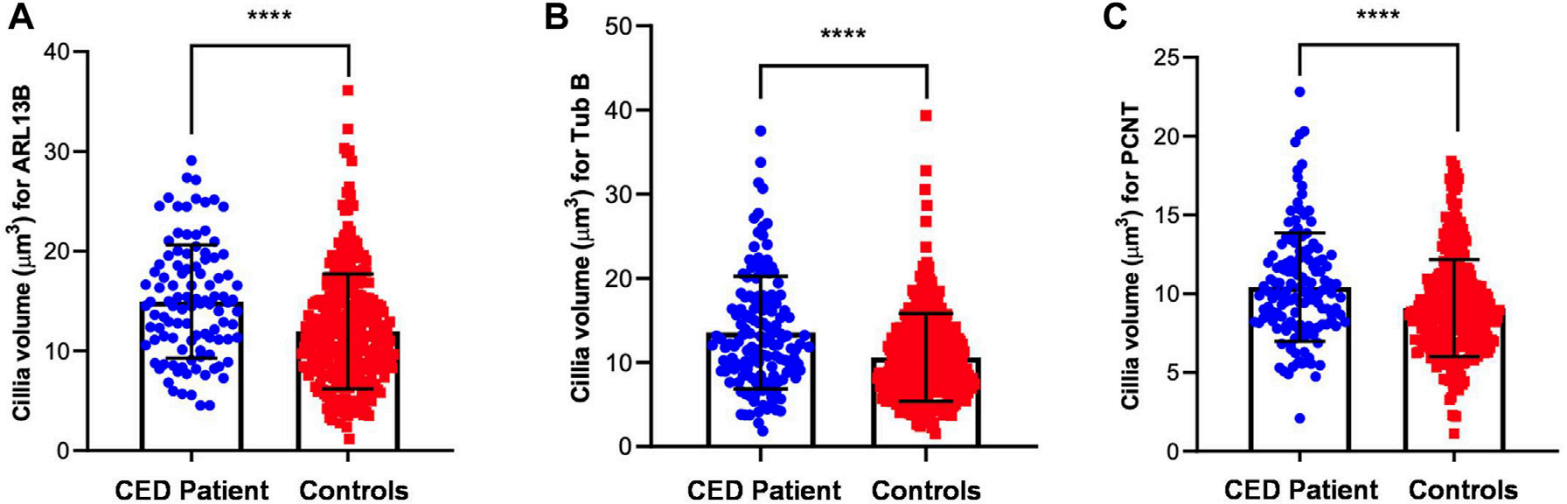
Volume measurements of ARL13B, Acet-Tub, and PCNT. The amount of all markers was calculated and revealed a highly significant increase in volume for all three proteins (Acet-Tub, ARL13B, and PCNT: *p* < 0.0001) **(A–C)**.

To provide additional evidence that the function of WDR35 was disturbed, we performed cilium phenotyping in hURECs from the patient and three unrelated and unaffected pediatric controls. Cilium frequency and morphology, including cilium length, height, and width, were evaluated by immunofluorescence (IF) experiments. Specific IF antibodies were used to visualize different ciliary compartments: acetylated-alpha-tubulin (Acet-Tub) for the ciliary axoneme, ADP-ribosylation factor-like protein 13B (ARL13B) for the ciliary membrane, and pericentrin (PCNT) to mark the base of the cilium (Figure 2B). No significant difference (ns) in ciliogenesis (percentage of ciliated cells) of the hURECs was detected when comparing the CED patient to the combined controls (Figure 2C). Interestingly, there was a statistical difference (*p* < 0.01) in ciliogenesis between the patient and control 1, the sex- and age-matched control (Figure 2D). Moreover, several differences in cilium morphology were observed while comparing the cilia from the CED patient to those of all three controls. The cilium morphology analysis included measurements of cilium width, height, and length using two markers visualizing the ciliary axoneme, namely, Acet-Tub and ARL13B. The cilia of the CED patient were significantly wider (Acet-Tub and ARL13B: *p* < 0.0001) and longer (Acet-Tub: *p* < 0.01 and ARL13B: *p* < 0.05) compared to those of the controls (Figures 3A, B, E, F). Evaluation of cilium height for both axonemal markers showed no differences between the patient and controls (Figures 3C, D). Combining these data as a measurement of the cilium volume showed a highly significant increase for all three measured proteins (Acet-Tub, ARL13B, and PCNT: *p* < 0.0001) (Figures 4A–C).

## 4 Discussion

In this study, we report on a 5-year-old female patient diagnosed with cranioectodermal dysplasia. The patient presented with a distinctive CED phenotype (Tan et al., 2021). A detailed clinical description has been provided. Interestingly, prenatal ultrasound examination showed the presence of increased NT at 12 weeks and 1 day of gestation. This finding is in agreement with previous literature reports suggesting that increased NT might be associated with CED (Walczak-Sztulpa et al., 2020; Walczak-Sztulpa et al., 2021). Genetic analysis revealed compound heterozygous variants in *WDR35*, the most frequently disrupted gene in CED-affected individuals (Walczak-Sztulpa et al., 2022). The detected variants are a previously described nonsense variant p. (Leu641*) in exon 18 and a novel missense variant p. (Ala1027Thr) in exon 26. The p. (Leu641*) variant is the most frequently reported variant in *WDR35* (Hoffer et al., 2013; Li et al., 2015; Zhang et al., 2018b; Brndiarova et al., 2021; Walczak-Sztulpa et al., 2021; Walczak-Sztulpa et al., 2022). Similar to the presented patient, the most frequent combination of variants among CED patients with *WDR35* defects is a combination of a missense and a loss of function (LOF) variant present in trans. To date, 22 missense variants have been described in the *WDR35* gene in patients with a ciliopathy-related phenotype, of which 13 changes were found in CED-affected individuals (HGMD, 11th October 2023). All described variants, including GnomAD allele frequencies, ClinVar interpretation, ACMG classification, CADD and AlphaMissense scores, and *in silico* predictions (AlphaMissense, SIFT, PolyPhen2, and MutationTaster), has been presented in Supplementary Table S1 (Ng and Henikoff, 2003; Adzhubei et al., 2010; Schwarz et al., 2014; Cheng et al., 2023). Among these variants, six were classified as likely pathogenic (LP), and 17 (including novel reported change) were classified as variants of uncertain significance (VUS) based on ACMG criteria (Richards et al., 2015), indicating that missense VUS changes are common in *WDR35*. Moreover, those changes are absent in GnomAD or have a very low population frequency. *In silico* analysis, including four algorithms, showed that 11 variants were predicted to be probably damaging for all predictors, and eight changes were assumed to be pathogenic using three prediction algorithms. The novel variant identified in this study, missense variant p. (Ala1027Thr), has been predicted to be deleterious by SIFT, PolyPhen2, and MutationTaster analysis. All missense VUS variants reported in the *WDR35* gene in patients with ciliopathy-related diseases have been located across the entire length of the WDR35 protein, shown in Supplementary Figure S2 (the figure was created using the proteinpaint website). Identification of the missense variant p. (Ala1027Thr), which has not been reported to date, expands the growing list of variants found in CED patients. In addition to the detected variants in *WDR35*, array CGH analysis of the patient’s DNA revealed the presence of a deletion located on chromosome 7q31.1, which was inherited from the apparently healthy mother. The deleted region spans exons 1 to 3 and part of intron 3 of *IMMP2L*. Loss of this part of the gene has been reported in various neuropsychiatric disorders, including autism spectrum disorder (ASD), psychomotor delay, Gilles de la Tourette syndrome (GTS), and a subtype of schizophrenia (SCZ) characterized by increased plasma pentosidine (PEN-SCZ) (Yoshikawa et al., 2022). Moreover, differential DNA methylation of *IMMP2L* in families with maternally inherited 7q31.1 deletions has been shown to be associated with intellectual disability and developmental delay (Vasilyev et al., 2021). Only one similar deletion detected in a patient with childhood apraxia of speech has been reported in the ClinVar database (ClinVar accession ID VCV000242956.1). However, a meta-analysis of 5,568 patients with ASD and 10,279 controls revealed no associations between microdeletion in *IMMP2L* and autism (Zhang et al., 2018b). Moreover, deletions located on chromosome 7q31.1 have also been reported in healthy individuals in the Database of Genomic Variants (DGV). To date, no likely pathogenic or pathogenic variants have been described in *IMMP2L*. Taken together, further clinical data and functional experiments are needed to verify the correlation between the detected microdeletion and the clinical phenotype of the CED patient. As kidney dysfunction is a major cause of morbidity and mortality in CED patients and is present in approximately 93% of affected individuals with *WDR35* variants, we decided to study hURECs from the presented CED patient diagnosed with stage-II CKD (Tan et al., 2021). Our preliminary results showed that hURECs from the CED patient present with a different cilium morphology variation as compared to controls, most likely due to the identified variants in *WDR35*. Analysis of the CED patient-derived URECs revealed a significant increase in cilium length, width, and volume compared to controls (based on data using Acet-Tub, ARL13B, and PCNT to visualize the cilium parameters). To our knowledge, this is the first report presenting cilium morphology abnormalities in hURECs from a CED patient with *WDR35* variants. It is known that the WDR35 protein belongs to the IFT-A complex, and thus, the observed abnormalities in the cilium phenotype of the CED patient might be associated with defective IFT machinery leading to the accumulation of proteins along the ciliary axoneme. Our findings are consistent with the results previously obtained from hURECs from a patient with a ciliopathy known as Joubert syndrome (JBTS). In this patient, pathogenic compound heterozygous variants in *CEP290* were identified. IF analysis and scanning electron microscopy (SEM) experiments showed abnormalities in the cilium phenotype of JBTS patients’ hURECs, including elongated and disorganized cilia (Srivastava et al., 2017). Furthermore, experiments performed in hURECs from a patient with autosomal recessive polycystic kidney disease (ARPKD) and the *PKHD1* variants revealed abnormally elongated cilia and the presence of multiple blebs along the axoneme (Molinari et al., 2020). Moreover, studies in hURECs obtained from a patient with Maizer–Saldino syndrome (MZSDS) and variants in *IFT140* showed an accumulation of the IFT88 protein, which is a component of IFT-B complex B. These results suggest defective retrograde IFT transport from the ciliary tip to the base of the cilium (Oud et al., 2018). Still, data concerning the cilia in hURECs from ciliopathy patients are limited, and further functional experiments are required to expand the knowledge in this field. It is known that the specific functioning of the primary cilia in the kidney plays a crucial role in the kidney’s organogenesis and differentiation, as well as the proliferation of renal epithelial cells. Defects of primary cilia are associated with renal diseases, often leading to ESRD, requiring kidney replacement therapy (Bai et al., 2022). Our results indicate that defects of primary cilia in renal epithelial cells may be associated with renal insufficiency present in the CED patient with *WDR35* variants. The combination of clinical examination, genetic testing, and functional studies plays an important role in understanding patients’ phenotypes. The diagnostic journey starts with the identification of the genetic defect and its effect on protein function, followed by verification at the cellular level, and in the end, ultimately leads to an explanation for the clinical phenotype. Urine provides a great source of patient-derived kidney cells obtained in a non-invasive way. These hURECs are a valuable model for studying renal diseases, especially those associated with cilia dysfunction, and can be used for the identification of potential therapeutic treatment.

## Data Availability

The datasets for this article are not publicly available due to concerns regarding participant/patient anonymity. Requests to access the datasets should be directed to the corresponding author.

## Supplementary material

SUPPLEMENTARY FIGURE S1 Data Sheet 1.

SUPPLEMENTARY FIGURE S2 Image 1.

## References

[B1] Adzhubei I. A. Schmidt S. Peshkin L. Ramensky V. E. Gerasimova A. Bork P. (2010). A method and server for predicting damaging missense mutations. Nat. Methods 7 (4), 248–249. 10.1038/nmeth0410-248 20354512 PMC2855889

[B2] Bai Y. Wei C. Li P. Sun X. Cai G. Chen X. (2022). Primary cilium in kidney development, function and disease. Front. Endocrinol. (Lausanne) 13, 952055. 10.3389/fendo.2022.952055 36072924 PMC9441790

[B3] Brndiarova M. Mraz M. Kolkova Z. Cisarik F. Banovcin P. (2021). Sensenbrenner syndrome presenting with severe anorexia, failure to thrive, chronic kidney disease and angel-shaped middle phalanges in two siblings. Mol. Syndromol. 12 (4), 263–267. 10.1159/000515645 34421506 PMC8339521

[B4] Caparros-Martin J. A. De Luca A. Cartault F. Aglan M. Temtamy S. Otaify G. A. (2015). Specific variants in WDR35 cause a distinctive form of Ellis-van Creveld syndrome by disrupting the recruitment of the EvC complex and SMO into the cilium. Hum. Mol. Genet. 24 (14), 4126–4137. 10.1093/hmg/ddv152 25908617 PMC4560068

[B5] Cheng J. Novati G. Pan J. Bycroft C. Žemgulytė A. Applebaum T. (2023). Accurate proteome-wide missense variant effect prediction with AlphaMissense. Science 22 (6664), eadg7492. 10.1126/science.adg7492 37733863

[B6] Hoffer J. L. Fryssira H. Konstantinidou A. E. Ropers H. H. Tzschach A. (2013). Novel WDR35 mutations in patients with cranioectodermal dysplasia (Sensenbrenner syndrome). Clin. Genet. 83 (1), 92–95. 10.1111/j.1399-0004.2012.01880.x 22486404

[B7] Li Y. Garrod A. S. Madan-Khetarpal S. Sreedher G. McGuire M. Yagi H. (2015). Respiratory motile cilia dysfunction in a patient with cranioectodermal dysplasia. Am. J. Med. Genet. A 167A (9), 2188–2196. 10.1002/ajmg.a.37133 25914204

[B8] McConnachie D. J. Stow J. L. Mallett A. J. (2021). Ciliopathies and the kidney: a review. Am. J. Kidney Dis. 77 (3), 410–419. 10.1053/j.ajkd.2020.08.012 33039432

[B9] Mill P. Christensen S. T. Pedersen L. B. (2023). Primary cilia as dynamic and diverse signalling hubs in development and disease. Nat. Rev. Genet. 24 (7), 421–441. 10.1038/s41576-023-00587-9 37072495 PMC7615029

[B10] Mill P. Lockhart P. J. Fitzpatrick E. Mountford H. S. Hall E. A. Reijns M. A. (2011). Human and mouse mutations in WDR35 cause short-rib polydactyly syndromes due to abnormal ciliogenesis. Am. J. Hum. Genet. 88 (4), 508–515. 10.1016/j.ajhg.2011.03.015 21473986 PMC3071922

[B11] Molinari E. Srivastava S. Dewhurst R. M. Sayer J. A. (2020). Use of patient derived urine renal epithelial cells to confirm pathogenicity of PKHD1 alleles. BMC Nephrol. 15 (1), 435. 10.1186/s12882-020-02094-z PMC7559414 33059616

[B12] Ng P. C. Henikoff S. (2003). SIFT: predicting amino acid changes that affect protein function. Nucleic Acids Res. 31 (13), 3812–3814. 10.1093/nar/gkg509 12824425 PMC168916

[B13] Oud M. M. Latour B. L. Bakey Z. Letteboer S. J. Lugtenberg D. Wu K. M. (2018). Cellular ciliary phenotyping indicates pathogenicity of novel variants in IFT140 and confirms a Mainzer-Saldino syndrome diagnosis. Cilia 7, 1. 10.1186/s13630-018-0055-2 30479745 PMC6247778

[B14] Pazour G. J. Quarmby L. Smith A. O. Desai P. B. Schmidts M. (2020). Cilia in cystic kidney and other diseases. Cell Signal 69, 109519. 10.1016/j.cellsig.2019.109519 31881326 PMC6953175

[B15] Richards S. Aziz N. Bale S. Bick D. Das S. Gastier-Foster J. ACMG Laboratory Quality Assurance Committee (2015). Standards and guidelines for the interpretation of sequence variants: a joint consensus recommendation of the American college of medical genetics and Genomics and the association for molecular pathology. Genet. Med. 17 (5), 405–424. 10.1038/gim.2015.30 25741868 PMC4544753

[B16] Schwarz J. M. Cooper D. N. Schuelke M. Seelow D. (2014). MutationTaster2: mutation prediction for the deep-sequencing age. Nat. Methods 11 (4), 361–362. 10.1038/nmeth.2890 24681721

[B17] Srivastava S. Ramsbottom S. A. Molinari E. Alkanderi S. Filby A. White K. (2017). A human patient-derived cellular model of Joubert syndrome reveals ciliary defects which can be rescued with targeted therapies. Hum. Mol. Genet. 26 (23), 4657–4667. 10.1093/hmg/ddx347 28973549 PMC5886250

[B18] Tan W. Lin A. Keppler-Noreuil K. (2021). “Cranioectodermal dysplasia,” in GeneReviews. Bethesda, MD: National Library of Medicine.

[B19] Vasilyev S. A. Skryabin N. A. Kashevarova A. A. Tolmacheva E. N. Savchenko R. R. Vasilyeva O. Y. (2021). Differential DNA methylation of the IMMP2L gene in families with maternally inherited 7q31.1 microdeletions is associated with intellectual disability and developmental delay. Cytogenet Genome Res. 161 (3-4), 105–119. 10.1159/000514491 33849037

[B20] Walczak-Sztulpa J. Wawrocka A. Leszczynska B. Mikulska B. Arts H. H. Bukowska-Olech E. (2020). Prenatal genetic diagnosis of cranioectodermal dysplasia in a Polish family with compound heterozygous variants in WDR35. Am. J. Med. Genet. A 182 (10), 2417–2425. 10.1002/ajmg.a.61785 32804427

[B21] Walczak-Sztulpa J. Wawrocka A. Sikora W. Pawlak M. Bukowska-Olech E. Kopaczewski B. (2022). WDR35 variants in a cranioectodermal dysplasia patient with early onset end-stage renal disease and retinal dystrophy. Am. J. Med. Genet. A 188 (10), 3071–3077. 10.1002/ajmg.a.62903 35875935

[B22] Walczak-Sztulpa J. Wawrocka A. Stanczyk M. Pesz K. Dudarewicz L. Chrul S. (2021). Interfamilial clinical variability in four Polish families with cranioectodermal dysplasia and identical compound heterozygous variants in WDR35. Am. J. Med. Genet. A 185 (4), 1195–1203. 10.1002/ajmg.a.62067 33421337

[B23] Yoshikawa A. Kushima I. Miyashita M. Suzuki K. Iino K. Toriumi K. (2022). Exonic deletions in IMMP2L in schizophrenia with enhanced glycation stress subtype. PLoS One 17 (7), e0270506. 10.1371/journal.pone.0270506 35776734 PMC9249242

[B24] Zhang W. Taylor S. P. Ennis H. A. Forlenza K. N. Duran I. Li B. (2018a). Expanding the genetic architecture and phenotypic spectrum in the skeletal ciliopathies. Hum. Mutat. 39 (1), 152–166. 10.1002/humu.23362 29068549 PMC6198324

[B25] Zhang Y. Liu Y. Zarrei M. Tong W. Dong R. Wang Y. (2018b). Association of IMMP2L deletions with autism spectrum disorder: a trio family study and meta-analysis. Am. J. Med. Genet. B Neuropsychiatr. Genet. 177 (1), 93–100. 10.1002/ajmg.b.32608 29152845

