# Supplementary material for: Ciliary phenotyping in renal epithelial cells in a cranioectodermal dysplasia patient with WDR35 variants
Source: Front Mol Biosci. 2023 Dec 12;10:1285790. doi: 10.3389/fmolb.2023.1285790 (PMC10756907; doi:10.3389/fmolb.2023.1285790)
Supplement: Supplementary file 1 [file Table1.DOCX]

|  | HGVS  (nucleotide) | HGVS  (protein) | GnomAD AF  Exomes | GnomAD AF  Genomes | ClinVar Interpretation | ClinVar  Accession# | ACMG  classification | CADD  score | AlphaMissense  score | AlphaMissense  prediction | SIFT | PolyPhen2 | MutationTaster | Reported phenotype | Reference |
| --- | --- | --- | --- | --- | --- | --- | --- | --- | --- | --- | --- | --- | --- | --- | --- |
| 1. | c.206G>A | p.(G69D) | Absent | Absent | Pathogenic | VCV000431796.22 | Likely pathogenic | 26,7 | 0,7516 | Pathogenic | Deleterious | Probably damaging | Deleterious | Cranioectodermal dysplasia, Hepatic cysts with ductal malformation, polycystic kidney with renal failure, skeletal dysplasia & cerebellar hypoplasia | Al. Noaim (2022) Horm Res Paediatr 96(4):426-431, Shaheen (2016) Genet Med 18, 686 |
| 2. | c.392G>T | p.(C131F) | Absent | 1,31E-05 | - | - | VUS | 28,7 | 0,9575 | Pathogenic | Deleterious | Probably damaging | Deleterious | Skeletal ciliopathy | Molina-Ramírez (2022) J Med Genet 59, 393 |
| 3. | c.504T>A | p.(S168R) | Absent | Absent | Likely pathogenic | VCV001480830.4 | VUS | 20,9 | 0,8532 | Pathogenic | Tolerated | Benign | Deleterious | Cranioectodermal dysplasia | Hoffer (2013) Clin Genet 83, 92 |
| 4. | c.622G>C | p.(A208P) | Absent | Absent | - | - | VUS | 26,6 | 0,8683 | Pathogenic | Tolerated | Possibly damaging | Deleterious | Nephronophthisis-related ciliopathy | Yamamura (2017) Clin Exp Nephrol 21, 136 |
| 5. | c.707G>A | p.(C236Y) | Absent | Absent | Pathogenic | VCV000872722.16 | Likely pathogenic | 24,2 | 0,7352 | Pathogenic | Deleterious | Possibly damaging | Deleterious | Cranioectodermal dysplasia | Li (2015) Am J Med Genet A 167, 2188 |
| 6. | c.781T>C | p.(W261R) | 3,98E-06 | 6,57E-06 | Pathogenic | VCV000031045.1 | Likely pathogenic | 25,8 | 0,9986 | Pathogenic | Deleterious | Probably damaging | Deleterious | Short rib-polydactyly syndrome | Mill (2011) Am J Hum Genet 88, 508 |
| 7. | c.794G>T | p.(G265V) | Absent | Absent | - | - | VUS | 24,9 | 0,9665 | Pathogenic | Deleterious | Probably damaging | Deleterious | Short rib-polydactyly syndrome | Gabriel (2022) Prenat Diagn 42, 845 |
| 8. | c.907G>A | p.(G303R) | 2,39E-05 | 2,63E-05 | - | - | VUS | 29,6 | 0,7579 | Pathogenic | Deleterious | Probably damaging | Deleterious | Cranioectodermal dysplasia | Walczak-Sztulpa (2022) Am J Med Genet A 188, 3071 |
| 9. | c.932G>T | p.(W311L) | 1,72E-03 | 1,37E-03 | Pathogenic | VCV000446644.16 | VUS | 27,8 | 0,9357 | Pathogenic | Deleterious | Probably damaging | Deleterious | Short-rib polydactyly syndrome | Toriyama (2016) Nat Genet 48, 648 |
| 10. | c.1183A>T | p.(N395Y) | Absent | Absent | VUS | VCV000288569.36 | VUS | 23,6 | 0,0887 | Benign | Tolerated | Benign | Deleterious | Short rib-polydactyly syndrome | Zhang (2018) Hum Mutat 39, 152 |
| 11. | c.1255G>A | p.(G419S) | Absent | Absent | - | - | VUS | 32 | 0,7003 | Pathogenic | Tolerated | Probably damaging | Deleterious | Nephronophthisis-related ciliopathy | Sakakibara (2022) J Hum Genet 67, 427 |
| 12. | c.1292C>G | p.(P431R) | 3,18E-05 | 3,29E-05 | - | - | VUS | 25,2 | 0,9685 | Pathogenic | Deleterious | Probably damaging | Deleterious | Nephronophthisis-related ciliopathy | Sakakibara (2022) J Hum Genet 67, 427 |
| 13. | c.1415G>A | p.(R472Q) | Absent | Absent | Likely pathogenic | VCV000437865.1 | VUS | 24,4 | 0,1188 | Benign | Tolerated | Possibly damaging | Deleterious | Cranioectodermal dysplasia | Córdova-Fletes (2018) Eur J Med Genet 61, 161 |
| 14. | c.1433G>A | p.(R478K) | Absent | Absent | - | - | VUS | 33 | 0,1054 | Benign | Tolerated | Benign | Deleterious | Short rib-polydactyly syndrome | Duran (2017) Cilia 6, 7 |
| 15. | c.1592T>C | p.(L531P) | Absent | 6,57E-06 | Pathogenic | VCV000065620.3 | Likely pathogenic | 28,2 | 0,9373 | Pathogenic | Deleterious | Probably damaging | Deleterious | Cranioectodermal dysplasia | Bacino (2012) Am J Med Genet A 158A, 2917 |
| 16. | c.1877A>G | p.(E626G) | Absent | Absent | Pathogenic | VCV000000021.3 | VUS | 35 | 0,8742 | Pathogenic | Tolerated | Probably damaging | Deleterious | Cranioectodermal dysplasia | Gilissen (2010) Am J Hum Genet 87, 418 |
| 17. | c.2522A>T | p.(D841V) | Absent | Absent | (Likely) Pathogenic | VCV000446645.2 | VUS | 28,1 | 0,7552 | Pathogenic | Tolerated | Probably damaging | Deleterious | Cranioectodermal dysplasia | Walczak-Sztulpa (2017) Am J Med Genet A 173, 1364 |
| 18. | c.2623G>A | p.(A875T) | 3,98E-06 | 6,57E-05 | Likely pathogenic | VCV000000023.10 | Likely pathogenic | 32 | 0,8382 | Pathogenic | Deleterious | Probably damaging | Deleterious | Cranioectodermal dysplasia | Gilissen (2010) Am J Hum Genet 87, 418 |
| 19. | c.2912A>G | p.(Y971C) | 7,96E-06 | Absent | - | - | VUS | 25,5 | 0,279 | Benign | Deleterious | Probably damaging | Deleterious | Cranioectodermal dysplasia | Hoffer (2013) Clin Genet 83, 92 |
| 20. | c.3079G>A | p.(A1027T) | Absent | Absent | - | - | VUS | 29,2 | 0,4475 | Ambiguous | Deleterious | Probably damaging | Deleterious | Cranioectodermal dysplasia | Present patient |
| 21. | c.3091C>T | p.(H1031Y) | Absent | Absent | VUS | VCV000488654.2 | VUS | 27,6 | 0,5047 | Ambiguous | Deleterious | Probably damaging | Deleterious | Cranioectodermal dysplasia | Lin (2013) Am J Med Genet A 161, 2762 |
| 22. | c.3203A>G | p.(Y1068C) | 7,96E-06 | 2,63E-05 | Likely pathogenic | VCV000440412.11 | VUS | 29,6 | 0,1693 | Benign | Deleterious | Probably damaging | Deleterious | Cranioectodermal dysplasia | Lin (2013) Am J Med Genet A 161, 2762 |
| 23. | c.3459G>T | p.(W1153C) | Absent | Absent | Pathogenic | VCV000488657.1 | Likely pathogenic | 32 | 0,7819 | Pathogenic | Deleterious | Probably damaging | Deleterious | Cranioectodermal dysplasia | Smith (2016) Am J Med Genet A 170, 760 |

WDR35 transcript NM_001006657.2

GnomAD version 3.0, accession date 11.10.2023
