# Supplementary figures and images for: Ciliary phenotyping in renal epithelial cells in a cranioectodermal dysplasia patient with WDR35 variants
Source: Front Mol Biosci. 2023 Dec 12;10:1285790. doi: 10.3389/fmolb.2023.1285790 (PMC10756907; doi:10.3389/fmolb.2023.1285790)

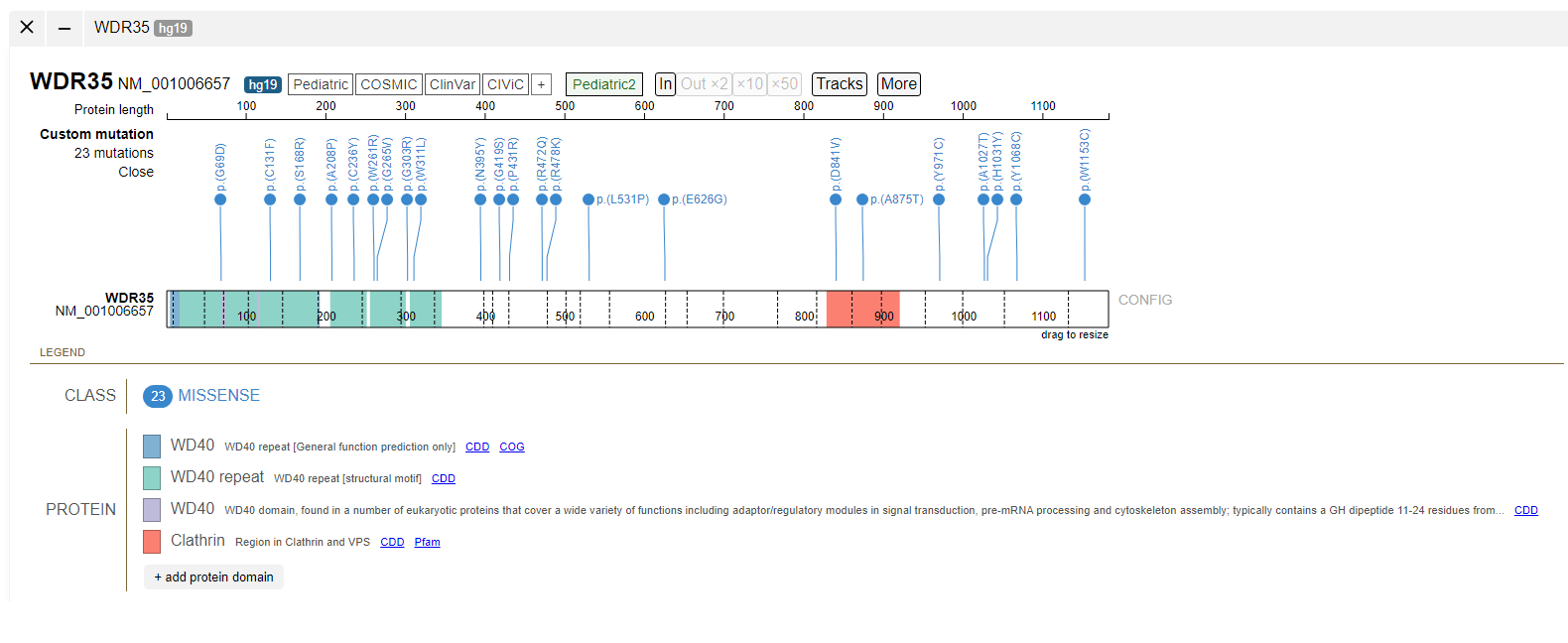

Supplement: Supplementary file 2 [file Image1.TIF]
