# Supplementary material for: Ciliary phenotyping in renal epithelial cells in a cranioectodermal dysplasia patient with WDR35 variants
Source: Front Mol Biosci. 2023 Dec 12;10:1285790. doi: 10.3389/fmolb.2023.1285790 (PMC10756907; doi:10.3389/fmolb.2023.1285790)
Supplement: Supplementary file 3 [file DataSheet1.PDF]

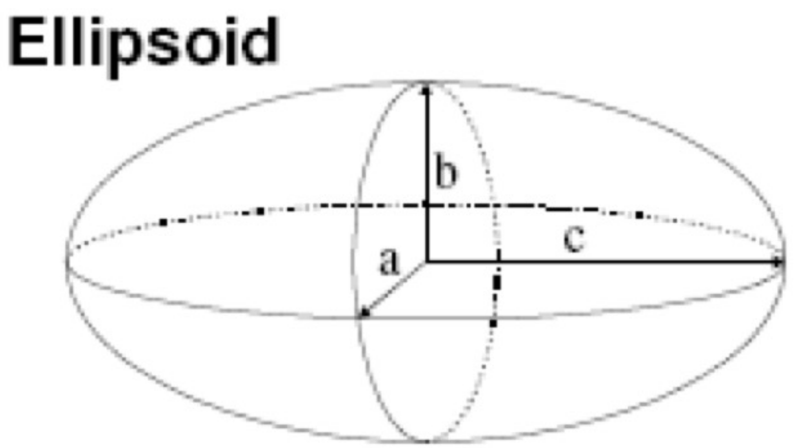

Cells - Cell Ellipsoid Axis A X, Y, Z
These three parameters define the Vector of the Ellipsoid Axis a.

Cells - Cell Ellipsoid Axis B X, Y, Z
These three parameters define the Vector of the Ellipsoid Axis b.

Cells - Cell Ellipsoid Axis C X, Y, Z
These three parameters define the Vector of the Ellipsoid Axis c.

Cells - Cell Ellipsoid Axis Length A, B, C
Cells - Cell Ellipsoid (oblate)
Cells - Cell Ellipsoid (prolate)

If:

$a \leq b \leq c$, then when:

- a = 0 it is an **Ellipse**
- a = b = c it is a **Sphere** (three equal sides)
- a ≠ b ≠ c it is a **scalene Ellipsoid** (three unequal sides)

http://www.bitplane.com/download/manuals/ReferenceManual9_2_0.pdf
